# Supplementary material for: Improved Reversion of Calcifications in Porcine Aortic Heart Valves Using Elastin-Targeted Nanoparticles
Source: Int J Mol Sci. 2023 Nov 17;24(22):16471. doi: 10.3390/ijms242216471 (PMC10671589; doi:10.3390/ijms242216471)
Supplement: Supplementary file 1 [file ijms-24-16471-s001.zip › ijms-2664797-supplementary.pdf]

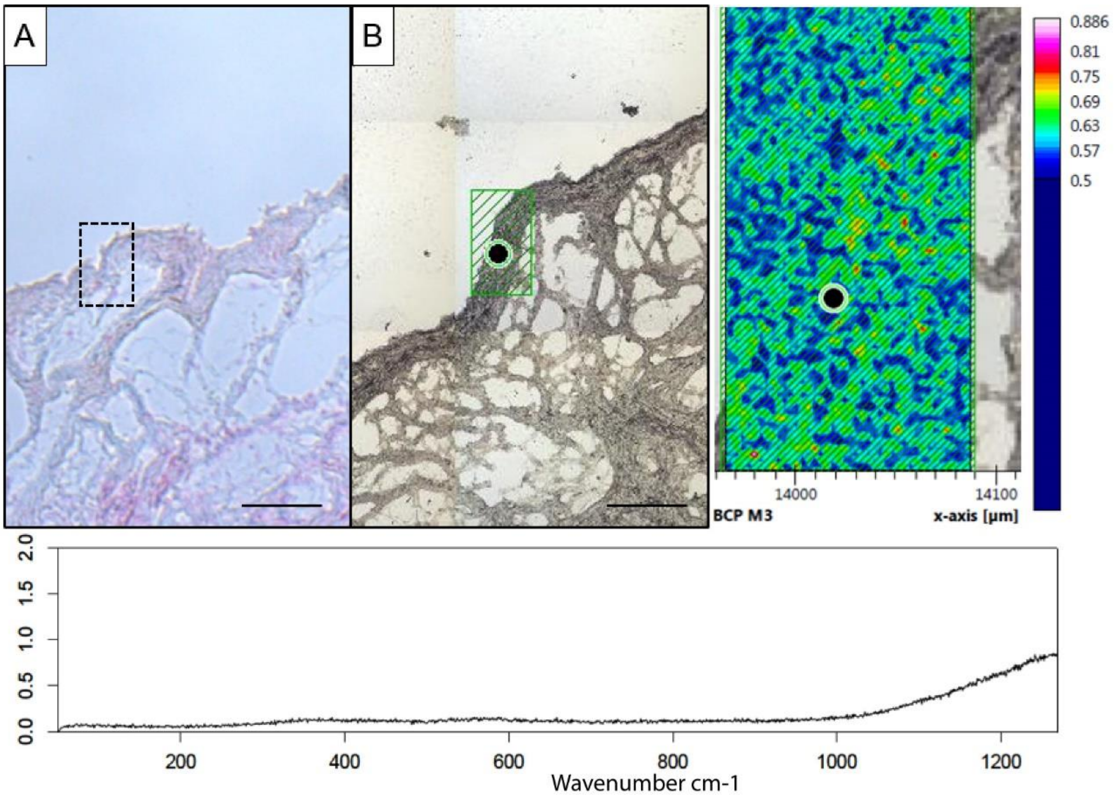

Figure S1: Representative Raman mapping of a non-calcified aortic valve cultured in standard medium for 10 days. A. Complementary von Kossa image displaying no calcification (left panel, scale bar: 200 µm). B. Representative overview image of an unstained, methanol-fixed, and air-dried aortic valve with selected ROI (left panel). Representative Raman spectrum displaying background noise with no peak for BCP at 960 cm^{-1} (lower panel, Y-axis shows the intensity of the scattered light (arbitrary units)). Representative heat map of BCP calcification after normalization and integration at 960 cm^{-1}, showing only background noise in the tissue (as determined by the integrated area under the curve at 945 – 975 cm^{-1}, right panel). n=1 valve leaflet, 4 sections per valve, 3-5 analyzed locations each.
